# Identification of the inferior alveolar canal using cone-beam computed tomography vs. panoramic radiography: a retrospective comparative study

**DOI:** 10.1186/s12903-023-03176-8

**Published:** 2023-07-04

**Authors:** Rawia Karameh, Mahmoud F. Abu-Ta’a, Khaled R. Beshtawi

**Affiliations:** Department of Dental Sciences, Faculty of Graduate Studies, Arab American University, Ramallah City, Palestine

**Keywords:** Inferior alveolar canal, CBCT, Panoramic, Visibility

## Abstract

**Background:**

This study aims at evaluating the visibility levels of the inferior alveolar canal (IAC) at different mandibular sites using panoramic (conventional & CBCT reformatted) and CBCT coronal views in a sample of a Palestinian population.

**Methods:**

The panoramic (conventional [CP] & CBCT reformatted [CRP]) and CBCT coronal views (CCV) of 103 patients (206 records, right and left sides) were analyzed. The visibility of IAC at five sites extending from the first premolar to the third mandibular molar region was evaluated visually (and compared among the radiographic views) as clearly visible, probably visible, invisible/poorly visible, or not present at the examined site. On CCV, the maximum dimension of the IAC (MD), the vertical distance (VD) between the mandibular cortex and IAC, and the horizontal position (HP) of the IAC were noted. Statistical significance in the differences and relationships of the variables was tested using several statistical tests.

**Results:**

There was a statistically significant relationship between the radiography modality (CP, CRP, CCV) and the visibility level of IAC (assessed in scores) at the five mandibular sites. When assessed on CP, CRP, and CCV, the IAC was clearly visible at all sites in 40.4%, 30.9%, and 39.6%, respectively, while being invisible/poorly visible in 27.5%, 38.9%, and 7.2% for the same views, respectively. The mean values of MD and VD were 3.61 mm and 8.48 mm, respectively.

**Conclusion:**

Different radiographic modalities would characterize the IAC’s structure in different qualities. Superior visibility levels were obtained interchangeably using CBCT cross-sectional views and conventional panorama at different sites compared to CBCT reformatted panorama. The IACs visibility was noted to improve at their distal aspects irrespective of the radiographic modality used. Gender —but not age— was a significant factor in the visibility level of IAC at only two mandibular sites.

## Introduction

The inferior alveolar canal (IAC) is a mandibular intraosseous structure that extends obliquely downward and forward in the ramus before running horizontally forward in the body of the mandible [1]. Identification of the IAC, including its location, course, morphology, and accessory branches, is essential as it contains vital structures and this aids in proper diagnosis and treatment planning [2, 3]. The preoperative assessment of the anatomical details of the IAC prior to multiple surgical interventions e.g., dental implants, fixations screws, and mandibular osteotomies would reduce the risk of injury to vital structures contained within the IAC [4].

Panoramic radiography [2, 5–7], conventional tomography [6], computed tomography (CT) [5, 8], and the more recently developed cone-beam computed tomography (CBCT) [3, 9], are all different radiographical modalities that have been investigated to evaluate the course of the IAC. CBCT imaging has several advantages over traditional two-dimensional imaging techniques, including eliminating the superimposition of nearby structures and preventing distortion [10].

The IAC is identified as a radiolucent zone with superior and inferior borders and is frequently visible on radiographs [2, 11]. The variability of IAC’s visibility on radiographs depends on the degree of cortication of its borders [2] and the trabecular quantity and arrangement around the IAC [11]. Although CBCT has been demonstrated to surpass traditional imaging modalities in depicting the IAC [2, 7], the visibility of this structure can vary greatly, even within the same person [12].

The current study aims at evaluating the visibility of the IAC on CBCT cross-sectional views, conventional panorama (CP), and CBCT reformatted panorama (CRP) in various mandibular sites among a sample population in the West Bank, Palestine. A review of the literature suggested that such studies have been not carried out on Palestinian inhabitants.

## Materials and methods

In this comparative, retrospective, cross-sectional study the radiographic records were retrieved from the database of patients who visited the dental centre at the Arab American University-Ramallah in Palestine, seeking general dental treatments between January 2018 and July 2022. This study was conducted after obtaining ethical clearance from the Palestinian health research council (# PHRC/HC/1090/22) and informed consent from the patients.

All the available data was initially analyzed and only the patients’ radiographs meeting the inclusion criteria were selected (non-randomized sampling). The inclusion criteria included dentulous patients of either gender (over 18 years of age), and patients who have both panoramic and CBCT volumes of diagnostic quality (taken at a maximum period of 6 months apart from each other). Patients presented with complete loss of teeth in the mandibular premolar-molar region (i.e., where the 1^st^, 2^nd^ premolars, and 1^st^ molar are missing) and/or jaw bone pathology in the region of interest, were excluded. Moreover, low-quality radiographs with distortion and technical errors were also excluded. Patients' gender and age were also noted. The panoramic radiographs were exposed using Sirona® XG5® (Dentsply Sirona®, Bensheim, Germany) where the CBCT volumes were acquired using the i-CAT™ FLX 17 (DEXIS™, Pennsylvania, USA) with exposure parameters: 64–73 kVp, 112 mAs, and 120 kVp, 5 mA, 4.8 s- 26.9 s, respectively. The CBCT volumes were saved in DICOM (digital imaging and communications in medicine) format and were analyzed by two examiners (i.e., the principal investigator and a maxillofacial radiologist) using the OnDemand® 3D Software (CyberMed®, Seoul, South Korea). The manufacturer's program Sidexis® 4 (Dentsply® Sirona®, Bensheim, Germany) was used to analyze the panoramic radiographs. The principal investigator performed the analysis and repeated it in full (2 weeks after the initial analysis), and the second examiner repeated it partly and independently. Prior to starting the study, each examiner had individual training to calibrate with the proposed methods. On a desktop-grade display, the radiographs were examined.

The IAC's visibility on radiographs was assessed in five mandibular regions: first premolar region (P1), second premolar region (P2), first molar region (M1), and second molar region (M2), and third molar region (M3). The periapical area inferior to the midline of root apices (in singular rooted teeth) and the mid-region between the mesial and distal roots (in multirooted teeth) were used as standardized regions for analysis. When only one tooth was missing between two present teeth, the mid-distance between those two teeth was used as a reference. Three radiographic views were used for this analysis i.e., conventional panorama (CP), CBCT reformatted panorama (CRP), and, CBCT coronal view (CCV). The CBCT reformatted panorama (CRP) was reconstructed using the “Auto-arch” function in the software and at the level of the mid-height of mandibular teeth roots. The CRP layer thickness was set at 22 mm with the sharpness filter set on “2x”.

**Fig. 1:**
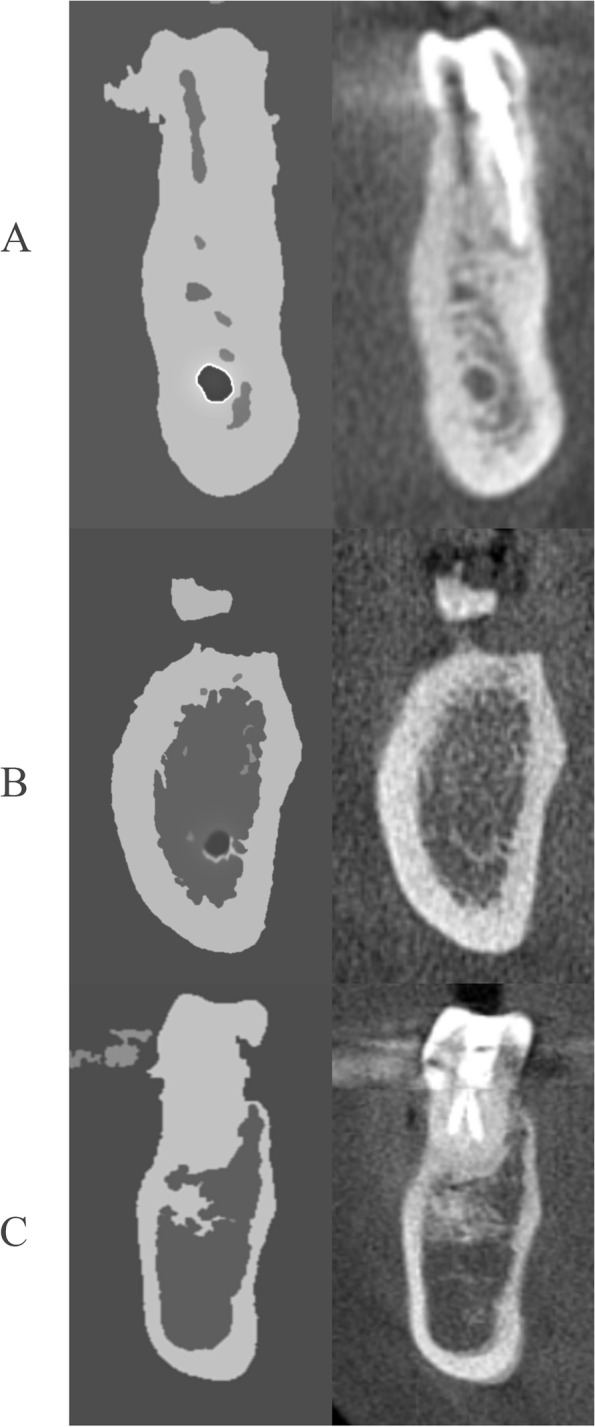
Evaluation methodology of the IAC’s visibility; diagrammatic (left) and corresponding CBCT coronal views (right) showing the various scores awarded (**A**, **B**, and **C**)

**Fig. 2:**
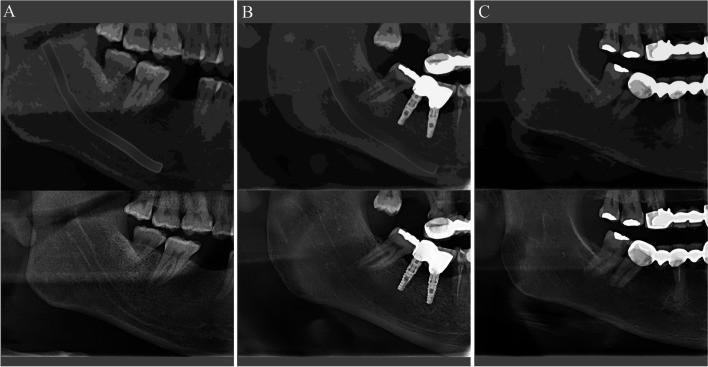
Evaluation methodology of the IAC’s visibility; diagrammatic (top) and corresponding panoramic radiograph (bottom) showing the various scores awarded (**A**, **B**, and **C**)

**Fig. 3:**
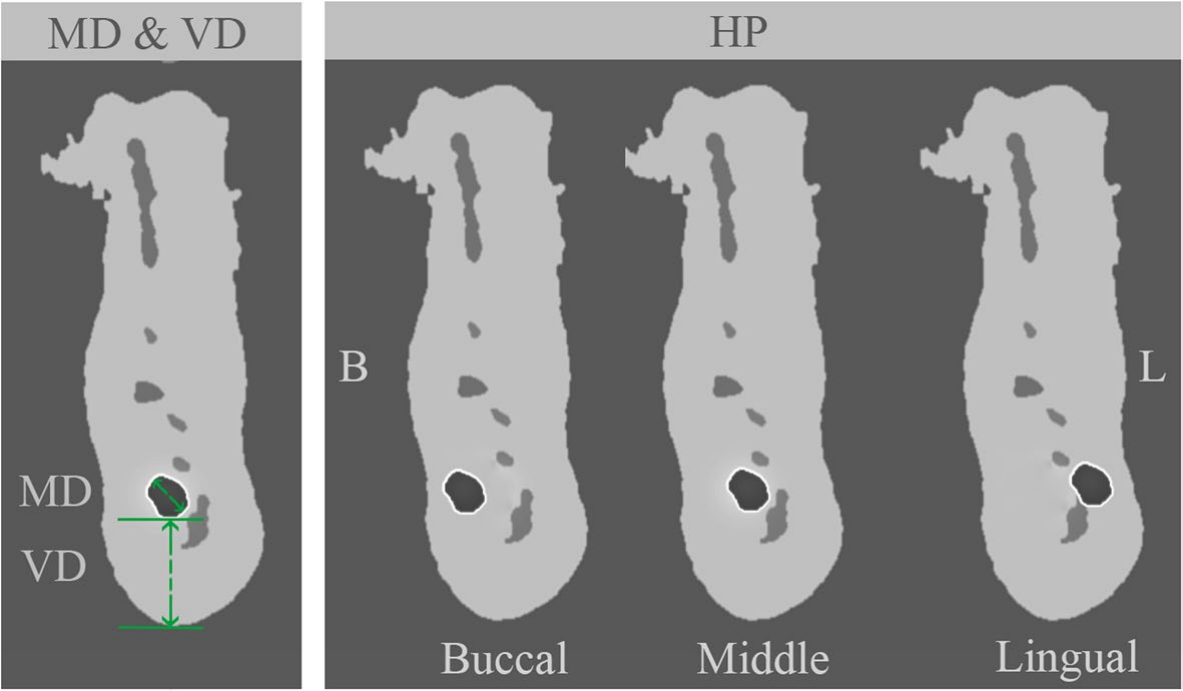
Diagrammatic images of CBCT coronal views show the methodology of measurements for the maximum dimension of the IAC (MD), vertical distance (VD), and horizontal position (buccal, lingual, and middle)

The visibility of the IAC on CBCT coronal view (CCV) was registered as A, B, C, and NP. Clearly visible canals were given the “A” score (i.e., well-defined and fully corticated), probably visible canals (i.e., moderately defined and partially corticated) were given the “B” score, and invisible/poorly visible canals (i.e., poorly defined and not corticated) were given the “C” score, while the “NP” (i.e., not present) score was given exactly at the site of, and/or mesial, to the mental canal where the IAC cease to exist/continue as the incisive canal (Fig. 1). The visibility of IAC on panoramic views (i.e., CRP and CP) was given the scores “A” if it was corticated and well-defined at its superior and inferior borders, the “B” score where only one border was corticated, and the “C” score where both of them were non-corticated and poorly defined (Fig. 2). The visibility scores of CRP, CP, and CCV were compared. The maximum dimension of the IAC (MD), the vertical distance (VD) between the inferior mandibular cortex to the inferior border of the IAC, and the horizontal position (HP: buccal, lingual, and middle) were noted only on the CCV views (Fig. 3).

All data were analyzed using SPSS (IBM Corp. Released 2019. IBM SPSS Statistics for Windows, Version 26.0. Armonk, NY: IBM Corp). Normality tests to ensure the normal distribution of the data set were performed. Multiple statistical tests were used to check for a significant relationship between variables (e.g., visibility level *vs.* modalities used, gender, and age) including Fisher's Exact and Chi-Square Tests. The independent sample t-test, One-Way analysis of variance test, Kruskal–Wallis test, Mann–Whitney U test, and Wilcoxon signed-rank were used to examine differences across measures (e.g., MD and VD between genders, age groups, and mandibular sides, i.e., right vs left). The tested factors were deemed statistically significant at a *p*-value < 0.05. The intraclass correlation coefficient (ICC), with values between 0.75 to 0.9 indicating high reliability and values greater than 0.90 indicating excellent reliability, was used to test intra- and interobserver reliability.

## Results

Two hundred and six records (i.e., 103 patients, right and left sides) were analyzed. The Patients were 65% males and 35% females with different age groups (5.8% [20–29 years], 46.6% [30 – 49 years], 47.6% [> = 50 years]). The type of used radiographic modality (i.e., CP, CRP, and CCV) showed a statistically significant relationship with the visibility level of IAC (in scores) at the five mandibular sites (Table 1, A). This means that the type of radiographic modalities is strongly related to the visibility level of the IAC, which indicates that different radiographic techniques would represent the IAC structure in different qualities.

**Table 1:** (A) The relationship of the visibility scores and the radiographic modality used i.e., conventional panorama (CP), CBCT reformatted panorama (CRP), and CBCT coronal view (CCV). (B) The relationship of the visibility scores (only on CBCT coronal view) and gender

	**A**				**B**		
	**CP %(count)**	**CRP %(count)**	**CCV %(count)**	***p*-value**	**Male %(count)**	**Female %(count)**	***p*-value**
**Visibility of P1**							
Clearly visible (A)	2.4 (5)	2.4 (5)	0.0 (0)	^†^0.000^**^	0.0 (0)	0.0 (0)	^†^0.124
Probably visible (B)	2.9 (6)	0.5 (1)	2.4 (5)		1.5 (2)	4.2 (3)	
Invisible/poorly visible (C)	49.0 (101)	51.9 (107)	3.9 (8)		2.2 (3)	6.9 (5)	
Not present (NP)	45.7 (94)	45.2 (93)	93.7 (193)		69.3 (129)	88.9 (64)	
**Visibility of P2**							
Clearly visible (A)	16.0 (33)	13.1 (27)	21.3 (44)	^†^0.000^**^	21.6 (29)	20.8 (15)	^‡^0.083
Probably visible (B)	14.6 (30)	7.8 (16)	17.0 (35)		14.9 (20)	20.8 (15)	
Invisible/poorly visible (C)	45.1 (93)	52.9 (109)	7.3 (15)		4.5 (6)	12.6 (9)	
Not present (NP)	24.3 (50)	26.2 (54)	54.4 (112)		59.0 (79)	45.8 (33)	
**Visibility of M1**							
Clearly visible (A)	50.5 (104)	37.4 (77)	48.0 (99)	^†^0.000^**^	49.3(66)	45.8 (33)	^†^0.007
Probably visible (B)	23.8 (49)	24.7 (51)	40.8 (84)		44.8 (60)	33.3 (24)	
Invisible/poorly visible (C)	24.7 (51)	37.9 (78)	10.2 (21)		5.2 (7)	19.5 (14)	
Not present (NP)	1.0 (2)	0.0 (0)	1.0 (2)		0.7 (1)	1.4 (1)	
**Visibility of M2**							
Clearly visible (A)	61.2 (126)	46.1 (95)	54.9 (113)	^†^0.000^**^	59.0 (79)	47.2 (34)	^‡^0.010^**^
Probably visible (B)	25.2 (52)	22.8 (47)	36.4 (75)		36.5 (49)	36.1 (26)	
Invisible/poorly visible (C)	13.6 (28)	31.1 (64)	8.7 (18)		4.5 (6)	16.7 (12)	
Not present (NP)	0.0 (0)	0.0 (0)	0.0 (0)		0.0 (0)	0.0 (0)	
**Visibility of M3**							
Clearly visible (A)	71.8 (148)	55.3 (114)	73.8 (152)	^†^0.000^**^	79.1 (106)	63.9 (46)	^‡^0.019^**^
Probably visible (B)	23.3 (48)	23.8 (49)	20.4 (42)		17.9 (24)	25.0 (18)	
Invisible/poorly visible (C)	4.9 (10)	20.9 (43)	5.8 (12)		3.0 (4)	11.1 (8)	
Not present (NP)	0.0 (0)	0.0 (0)	0.0 (0)		0.0 (0)	0.0 (0)	

^†^Fisher's exact test

^‡^Chi-square test

^**^Statistically significant (*p*-value < 0.05). P1-2: 1^st^ and 2^nd^ premolar sites, M1-3: 1^st^, 2^nd^, 3^rd^ molars sites

In the P1 site on CCV, 93.7% of the IAC was not present (NP) compared to 45.7% & 45.2% for both panoramic views (CP&CRP) for the same visibility score, where 49% & 51.9% were invisible on the same panoramic views compared to only 3.9% invisible/poorly visible on CCV. The P2 site was mostly not present on CCV (54.4%), while invisible/poorly visible on CP (45%) and CRP (52.9%). The M1 site was most clearly visible on CCV (48%) and probably visible (40.8%) compared to 50.5% and 37.4% clearly visible on CP & CRP, respectively. The M2 site was clearly visible on the majority of the sample on CP (61.2%), CRP (46.1%), and CCV (54.9%). Finally, the M3 site was clearly visible in the majority of the three views i.e., CP (71.8%), CRP (55.3%), and CCV (73.8%), with being the least site to show invisible/poorly visible score among all sites. Moreover, the statistical analysis showed a significant relationship between gender and visibility level only at M2 & M3 sites (Table 1, B). The males showed significantly higher percentages with clearer visibility scores at those two sites than females. The relationship between the visibility scores at all sites and the age of the patients showed no statistical significance.

The mean maximum dimension of IAC (MD) and vertical distance (VD) for all the mandibular sites (P1, P2, M1, M2, and M3) were 3.61 mm and 8.48 mm, respectively. Statistical significance was found comparing the differences in the means of MD, VD, and -in addition- the relation of the horizontal position (HP) between all the mandibular sites analyzed (Table 2). The significant difference was denoted mainly in the MD difference between the sites P2 and M2. For the VD, the significant difference was mainly denoted between the sites P2&M1, and P2&M2, and also between the sites M3&M1 and M3&M2.

**Table 2:** Measurements’ differences of the maximum dimension of IAC (MD), vertical distance (VD), and horizontal position (HP) relationship compared for different anatomical sites

	**P1** (Mean ± SD)	**P2** (Mean ± SD)	**M1** (Mean ± SD)	**M2** (Mean ± SD)	**M3** (Mean ± SD)	***p*-value**
**MD**	2.99 ± 1.0	3.34** ± **0.78^a^	3.50** ± **0.96^ab^	3.79** ± **1.06^b^	4.43** ± **1.25^ab^	^‡^0.000^**^
**VD**	9.23** ± **1.84	8.34** ± **1.72 ^cb^	7.69** ± **1.85^a^	7.87** ± **1.88^a^	9.29** ± **2.56^c^	^‡^0.000^**^
**HP**	**%(count)**	**%(count)**	**%(count)**	**%(count)**	**%(count)**	
M	40.0 (2)	69.6 (55)	37.0 (68)	15.9 (30)	28.9 (56)	^†^0.000^**^
B	0.0 (0)	0.0 (0)	1.00 (2)	0.5 (1)	1.00 (2)	
L	60.0 (3)	30.4 (24)	62.0 (114)	83.6 (158)	70.1 (136)	

Data were retrieved from CBCT coronal views (CCV)

^‡^Kruskal–Wallis test (different letters within a row indicate a significant difference at the level of 5%)

^†^Fisher's exact test

^**^Statistically significant (*p*-value < 0.05). P1-2: 1^st^ and 2^nd^ premolar sites, M1-3: 1^st^, 2^nd^, 3^rd^ molars sites, M: middle, B: buccal, L: lingual

A comparison of the mean values difference of MD, VD, and HP (relationship and not difference) between the right and left sides of the same patient only showed a statistically significant difference in the MD of the M2 site and a significant relationship with the horizontal position of M3 (Table 3, A&B). Comparison of the same variables but for different age groups, only showed significance in the vertical distance (VD) of P2, M2 (mainly denoted between age groups II&III), M3 (mainly denoted between age categories I&III, II&III), (Table 4, A&B). Moreover, a statistically significant relationship was found between the horizontal position (HP) of the M1 site and the different age categories tested. Measurements’ differences of MD &VD compared between males and females showed only statistical difference in MD at the M2 site, and VD at P2&M1&M2&M3 with the males having more mean values (i.e., MD, & VD) than the females at these sites (Table 5).

**Table 3:** Measurements’ differences of the maximum dimension of IAC (MD), vertical distance (VD), and horizontal position (HP) relationship compared for right vs. left sides

**A**	**Right** (Mean ± SD)	**Left** (Mean ± SD)	***p*-value**
**Maximum dimension (MD)**			
P1	-	-	
P2	3.24** ± **0.79	3.37** ± **0.86	^‡^0.702
M1	3.52 ± 0.86	3.52 ± 1.16	^‡^0.365
M2	3.62** ± **1.17	3.91** ± **1.06	^‡^0.023**
M3	4.29** ± **1.27	4.56** ± **1.29	^‡^0.137
**Vertical distance (VD)**			
P1	-	-	
P2	8.58** ± **1.65	8.24** ± **1.83	^‡^0.392
M1	7.83** ± **1.95	7.54** ± **1.77	^‡^0.177
M2	7.96** ± **1.87	7.86** ± **2.03	^‡^0.789
M3	9.26** ± **2.61	9.34** ± **2.51	^‡^0.476
**B**	**Right %(count)**	**Left %(count)**	***p*-value**
**P1**			^†^1.000
M	50.0 (2)	0.0 (0)	
B	0.0 (0)	0.0 (0)	
L	50.0 (2)	100.0 (1)	
**P2**			^§^0.790
M	71.1 (27)	68.3 (28)	
B	0.0 (0)	0.0 (0)	
L	28.9 (11)	31.7 (13)	
**M1**			^†^0.730
M	40.2 (37)	33.7 (31)	
B	1.1 (1)	1.1 (1)	
L	58.7 (54)	65.2 (60)	
**M2**			^†^1.000
M	16.0 (15)	15.8 (15)	
B	0.0 (0)	1.1 (1)	
L	84.0 (79)	83.1 (79)	
**M3**			^†^0.045^**^
M	21.7 (21)	36.1 (35)	
B	1.0 (1)	1.0 (1)	
L	77.3 (75)	62.9 (61)	

Data were retrieved from CBCT coronal views (CCV)

^‡^Wilcoxon signed-rank test. (-): the test can’t be computed, since the number of observations is less than 2

Data were retrieved from CBCT coronal views (CCV)

^†^Fisher's exact test

^§^Chi-square test

^**^Statistically significant (*p*-value < 0.05). P1-2: 1^st^ and 2^nd^ premolar sites, M1-3: 1^st^, 2^nd^, 3^rd^ molars sites, M: middle, B: buccal, L: lingual

**Table 4:** Measurements’ differences of the maximum dimension of IAC (MD), vertical distance (VD), and horizontal position (HP) relationship in different age groups

**A**	**Group I** mean (± SD)	**Group II** mean (± SD)	**Group III** mean (± SD)	**Group IV** mean (± SD)	***p*-value**
**Maximum dimension (MD)**					
P1	-	-	2.60 ± 0.68	3.24 ± 1.29	^†^0.520
P2	3.07 ± 1.09	3.56 ± 0.70	3.23 ± 0.68	3.42 ± 0.87	^†^0.538
M1	3.39 ± 0.72	3.52 ± 0.66	3.36 ± 0.65	3.63 ± 1.23	^‡^0.631
M2	4.07 ± 0.93	3.76 ± 0.76	3.49 ± 0.72	3.98 ± 1.30	^‡^0.056
M3	4.76 ± 1.06	4.32 ± 1.09	4.11 ± 1.03	4.66 ± 1.41	^‡^0.067
**Vertical distance (VD)**					
P1	-	-	9.66 ± 2.72	8.95 ± 1.65	^†^0.782
P2	10.28 ± 0.83^a^	9.13 ± 1.58^a^	7.90 ± 1.81^a^	8.42 ± 1.54^a^	^†^0.041^**^
M1	8.54 ± 1.53	8.34 ± 1.92	7.58 ± 1.97	7.46 ± 1.70	^⊺^0.079
M2	8.73 ± 1.69^ab^	8.88 ± 1.68^a^	7.34 ± 2.02^b^	7.85 ± 1.69^ab^	^⊺^0.0001^**^
M3	10.36 ± 1.71^a^	10.15 ± 2.34^a^	8.51 ± 2.40^b^	9.46 ± 2.70^ab^	^⊺^0.003^**^
**B**	**Group I** **%(count)**	**Group II** **%(count)**	**Group III** **%(count)**	**Group IV** **%(count)**	***p*-value**
**P1**					^§^1.000
M	0.0 (0)	0.0 (0)	50.0 (1)	33.3 (1)	
B	0.0 (0)	0.0 (0)	0.0 (0)	0.0 (0)	
L	0.0 (0)	0.0 (0)	50.0 (1)	66.7 (2)	
**P2**					^§^1.000
M	66.7 (2)	66.7 (6)	71.4 (25)	68.7 (22)	
B	0.0 (0)	0.0 (0)	0.0 (0)	0.0 (0)	
L	33.3 (1)	33.3 (3)	28.6 (10)	31.3 (10)	
**M1**					^§^0.004^**^
M	54.5 (6)	19.2 (5)	49.2 (32)	30.5 (25)	
B	9.1 (1)	0.0 (0)	1.6 (1)	0.0 (0)	
L	36.4 (4)	80.8 (21)	49.2 (32)	69.5 (57)	
**M2**					^§^0.056
M	18.2 (2)	19.2 (5)	21.5 (14)	10.3 (9)	
B	9.1 (1)	0.0 (0)	0.0 (0)	0.0 (0)	
L	72.7 (8)	80.8 (21)	78.5 (51)	89.7 (78)	
**M3**					^§^0.197
M	33.3 (4)	19.2 (5)	39.4 (26)	23.3 (21)	
B	0.0 (0)	0.0 (0)	0.0 (0)	2.2 (2)	
L	66.7 (8)	80.8 (21)	60.6 (40)	74.5 (67)	

Data were retrieved from CBCT coronal views (CCV)

^‡^ Kruskal–Wallis test (different letters within a row indicate a significant difference at the level of 5%)

^†^Independent sample t-test

^⊺^One-Way Analysis of Variance test. (-): the test can’t be computed, since the number of observations is less than 2

^**^Statistically significant (*p*-value < 0.05)

Data were retrieved from CBCT coronal views (CCV)

^§^Fisher's Exact Test

^**^Statistically significant (*p*-value < 0.05). Age groups: Group I (20–29 years), Group II (30–39 years), Group III (40–49 years), Group IV (> = 50 years). P1-2: 1^st^ and 2^nd^ premolar sites, M1-3: 1^st^, 2^nd^, 3^rd^ molars sites, M: middle, B: buccal, L: lingual

**Table 5:** Measurements’ differences of the maximum dimension of IAC (MD), and vertical distance (VD) between males and females

	**Female** (Mean ± SD)	**Male** (Mean ± SD)	***p*-value**
**Maximum dimension (MD)**			
P1	3.24 ± 1.29	2.60 ± 0.68	^†^0.575
P2	3.34 ± 0.66	3.34 ± 0.85	^†^0.976
M1	3.48 ± 1.15	3.52 ± 0.86	^‡^0.404
M2	3.66 ± 1.38	3.85 ± 0.87	^‡^0.015^**^
M3	4.29 ± 1.26	4.51 ± 1.24	^‡^0.140
**Vertical distance (VD)**			
P1	8.96 ± 1.65	9.66 ± 2.72	^†^0.733
P2	7.58 ± 1.06	8.81 ± 1.88	^†^0.000^**^
M1	6.85 ± 1.28	8.06 ± 1.94	^‡^0.000^**^
M2	7.04 ± 1.42	8.26 ± 1.94	^‡^0.000^**^
M3	8.66 ± 2.21	9.60 ± 2.67	^‡^0.015^**^

Data were retrieved from CBCT coronal views (CCV)

^†^Independent sample t-test

^‡^Mann–Whitney U test

^**^Statistically significant (*p*-value < 0.05). P1-2: 1^st^ and 2^nd^ premolar sites, M1-3: 1^st^, 2^nd^, 3^rd^ molars sites

The intraclass correlation coefficient (ICC) showed excellent intra- and interobserver agreement. The inter-observer ICC determined for the measure score was 0.986 (95% CI: 0.983 to 0.988). The intra-observer ICC for the measure was 0.988 (95% CI: 0.986 to 0.990).

## Discussion

Comprehensive planning is the foundation of an effective dental treatment, which uses imaging to aid in diagnosis [13]. Radiographic examinations are crucial to a successful treatment plan [13]. The height and width of the bone, the degree of corticalization, the density of mineralization, and the amount of cancellous bone should all be considered during the preoperative assessment of surgical interventions involving the posterior mandible [14, 15]. However, because the two-dimensional image does not provide detailed diagnostic information about the relationship of anatomical structures, panoramic radiography is a supplementary examination that is initially requested before implant surgery but a more sophisticated imaging is required to increase intra-operative safety [16].

This study, in which the visibility of the inferior alveolar canal was assessed and compared on CBCT cross-sectional volumes and panoramic views (conventional and CBCT reformatted panoramic), is the first of its kind in Palestine. Accurate identification of IAC and the understanding of the capabilities of the available radiographic modalities to precisely present the structure is indispensable prior to multiple dental procedures in its vicinity.

### CBCT versus panoramic visibility

Although the IAC is identified as a radiolucent band surrounded by a cortical border, the degree of cortication affects the easiness of the identification of the structure on the radiographs [12].

IAC at the 1^st^ molar region on the cross-sectional CBCT volumes was shown to exhibit cortical borders in 59% [17]. Twenty-three percent of the IAC were still detected but without cortication, only 18% of the IAC weren’t detectable [17]. The Authors [17] also found that in the submandibular gland fossa region, the trabeculation pattern was strongly correlated with the cortication of the IAC.

CBCT reformatted panoramic (CRP) versus conventional digital panoramic radiographs (CP) were compared for their visibility levels of IAC at three sites [2]. The authors [2] found that the visibility levels of IAC detected on CRP were clearer than CP regardless of the examined site of IAC. At the same time, the third molar site was “best” rated in terms of visibility than the other sites [2].

A study by Jung et al. [18] showed that 22.7%,11.8%, and 1.3% of IAC at the first molar, 2^nd^ molar, and 3^rd^ molar regions – respectively- were invisible compared to 8.2%,5.7%, and 0.2% for the same sites but on CBCT cross-sectional views. They also concluded that the visibility level of IAC at the 1^st^ molar sites was inferior to that of the third molar ones. The CBCT was shown to provide better visibility levels of IAC than panoramic radiographs [18].

The visibility of the IAC was studied in six mandibular sites on cross-section CBCT views by Oliveira-Santos et al. [12]. The authors [12] found that 53% of the samples were “easy” to identify, whereas 25% and 22% of the sample were “difficult” and “very difficult” to identify, respectively.

Alkhader & Jarab [19] assessed the visibility of IAC (on cross-section CBCT) at the impacted mandibular third molar sites where they found that most sites showed “very good to excellent” visibility levels.

A study by Jameel et al. [20] compared the visibility scores (i.e., clear or unclear) of the IAC at 4 mandibular sites between panoramic and CBCT cross-sectional views. The authors [20] concluded that the CBCT showed a higher degree of IAC visibility scores compared to panoramic radiographs. The visibility of IAC was higher at 3^rd^ molar sites (73%) on panoramic views, whereas on CBCT it was higher in the premolar region (65%) and decreased moving distally. Gender did not affect the visibility levels of IAC [20].

The visibility of the superior and inferior borders of the IAC was compared between panoramic radiographs and medical CT scans [21]. The visibility scores were significantly higher for upper and lower IAC borders in CT scans compared to panoramic radiographs [21]. While the visibility scores of the lower border of IAC were higher than the superior border in all tested regions (in both panoramic and CT) [21].

Compared with the current analysis, the radiographic view used to assess the visibility of IAC was shown to be related to the visibility level revealed. This implies that using different radiographic modalities would characterize the IAC in different qualities. The statistically significant visibility levels were in favour of CCV (compared to CP &CRP) at M3 & P2 sites, and in favour of CP at M1 & M2 sites. At the P1 site, the CCV showed better identification of the non-present of the IAC at that site compared to CRP & CP. The mean percentage of all sites (P1-M3) were clearly visible in 40.4%,30.9%, and 39.6% analyzed on CP, CRP, and CCV, respectively, whereas being invisible in 27.5%,38.9%, and 7.2% of the sites for the same views, respectively. Interestingly, among this Palestinian sample, the conventional panorama and CCV were superior to CBCT reformatted panorama. The more posterior the IAC course, the more visibility levels were found regardless of the used radiographic modality. Notably at the P1 site, the IAC was evaluated as “Not present (NP)” in 93.7%, 45.7 and 45.2% in CCV, CP, and CRP, respectively. This implies that the CCV was superior to both CP & CRP in terms of identification of the mental canal and its mesial region (a termination of IAC) compared with CP and CRP where the scores were indecisive i.e., invisible/poorly visible. The benefit of the implication of CBCT cross-sectional views in the mental foramen region is thus appreciated.

### Relation of age and gender to the visibility of the canal

Inconsistent evidence was found in the literature regarding the relation of age and gender to the visibility of the canal. Kubiliuse et al. [22] and Oliveira-Santos et al. [17] were unable to identify any relationship between gender or age and visibility. This contrasts with Iwanaga et al. [23], Iwanaga et al. [24], and Miles et al. [15] who reported the opposite, however, without identifying the cause. According to Kamrun et al. [21], the reason could be that as people age, the visibility of IAC diminishes as a result of osteoporotic changes in the alveolar bone. The findings of Iwanaga et al. [23, 24] lend more support to this thesis. According to their research, more females than males had osteoporotic mandibles; as a result, when the canal cannot be seen on CBCT, the mandible is more likely to be in the osteoporotic group than the other groups. In the current analysis, gender -but not age- showed a statistically significant relation with visibility levels only at M2 & M3 sites which were in favour of males.

### The IAC maximum dimension (MD), horizontal plane (HP), and vertical distance (VD)

Regarding the vertical distance (VD) between the base of the mandible and the inferior border of the IAC, Lindh et al. [25] mentioned that measuring this distance was unchallenging as the canal’s inferior borders and the mandibular base are better detected than their superior counterpart borders. Additionally, bone atrophy and resorption do not have a substantial impact on the region of the mandible that runs from the inferior border of the canal to the bottom of the mandible.

In a sample of a Finnish population, the IAC's diameter was shown to be 2.1 mm_(Avg.)_ [1.2–3 mm] by Ylikontiola et al. [26]. The distance between IAC and the inferior mandibular cortex was 8 mm (left side) & 8.2 mm (Right side) posteriorly and 7.1 mm (left side) & 6.5 mm (right side) anteriorly in the IAC course [26]. Yu & Wong [27] in Taiwan, found the mean distance of IAC to the inferior mandibular cortex at the 2^nd^molar site to be 7.6 ± 1.69 mm [3.3 mm -12 mm]. Kilic et al. [28] in Turkey, reported a mean vertical distance (VD) of 10.52 ± 1.7 mm and a mean maximum diameter (MD) of the IAC of 2.52 mm. In a study on an Australian population, Yeh et al. [29] concluded that the range of the distance between the inferior border of the canal and the lower border of the mandible in all sites was 7.26 mm_(Avg.)_.

In the current study, the comparison of the MD, VD, and the HP (relationship and not difference) between the respective regions P1-M3 showed statistically significant differences. The M2 site was the only source of significance comparing the MD between the right and left sides, while significant relation of HP was only revealed at the M3 site. Regarding the VD, comparison between age groups showed significant results, particularly at P2, M2, and M3 sites where -in most- Group I (20–29 years) and Group II (30–39 years) showed higher VD means than age groups III &IV (40–49 and >  = 50 years). The males showed higher mean values and statistically significant differences than females in MD, & VD mean values at selected sites. The averages of MD and VD in the current Palestinian sample (at all sites) were 3.61 mm [2.99 – 4.43 mm] and 8.48 mm [7.69–9.29 mm], respectively. This MD value was comparable with a Sudanese sample (3.4 mm) [30] but was slightly higher compared to the Finnish [26] and Turkish [28] populations. The VD was comparable with the other studies [26, 27, 29] but lower than the study by Kilic et al. [28]. The majority of the IAC’s horizontal position was lingually situated at the M1, M2, and M3 sites, whereas being more in the middle at the P2 site.

### Limitations

The perception of the visibility level of IAC may be influenced by the radiographic exposure parameters (and subsequently the resultant quality) and to some extent the examiner's experience or personal judgment. Further research is required to determine the effect of these variables on the radiographic interpretation of the structure.

## Conclusions

Different radiological modalities might define the IAC structure in various ways. Superior visibility levels were attained by employing CBCT cross-sectional views and conventional panorama interchangeably at different sites compared to CBCT reformatted panorama. Regardless of the radiological modality utilized, the visibility of the IACs was seen to improve in their distal aspects. Gender, but not age, was a significant determinant in the visibility of IAC at two mandibular locations. The studied Palestinian sample was comparable/slightly above average in terms of maximum dimension and vertical distance of IAC compared to other populations.

## Data Availability

All data generated or analysed during this study are included in this published article.
